# Beet Root Peel Extract as a Natural Cost‐Effective pH Indicator and Food Preservative in Edible Film: Shelf Life Improvement of Cold‐Stored Trout Fillet

**DOI:** 10.1002/fsn3.4605

**Published:** 2024-11-15

**Authors:** Fatemeh Ghanbar Soleiman Abadi, Behnaz Bazargani‐Gilani, Aryou Emamifar, Alireza Nourian

**Affiliations:** ^1^ Department of Food Hygiene and Quality Control, Faculty of Veterinary Medicine Bu‐Ali Sina University Hamedan Iran; ^2^ Department of Food Science and Technology, College of Food Industry Bu‐Ali Sina University Hamedan Iran; ^3^ Department of Pathobiology, Faculty of Veterinary Medicine Bu‐Ali Sina University Hamedan Iran

**Keywords:** beetroot peel extract, chitosan, polyvinyl alcohol, silver nanoparticles, spoilage indicator, trout fillet

## Abstract

In this study, chitosan (C)‐polyvinyl alcohol (P) edible film containing bio‐fabricated nanosilver particles (nAg) (as antimicrobial agent) and beetroot peel extract (BRPE) (as antioxidant agent and pH indicator) was used as spoilage indicator in cold‐stored rainbow trout fillets. DPPH (2,2‐diphenyl‐1‐picrylhydrazyl) radical scavenging activity (43.02%), reducing power (2.87), and total phenolic content (360.50 mg GAE/g) of ethanolic BRPE were higher than aqueous extract. Silver nanoparticles were biosynthesized using silver nitrate reduction by chitosan, confirmed by UV–Visible spectroscopy, optical and scanning electron microscope images, and X‐ray diffraction analysis. The highest tensile strength (4.20 MPa) and elongation at break (118%) belonged to the CP‐BRPE film, and the lowest water vapor permeability (2.45 10^−5^ g/s/m/P) was related to the CP‐nAg film. Also, the lowest total viable count (6.17 log CFU/g), psychrotrophic bacteria (6.27 log CFU/g), *Enterobacteriaceae* (4.9 log CFU/g), pH (5.66), total volatile basic‐nitrogen (TVB‐N) (22.1 mg/100 g of fish), and thiobarbituric acid reactive substances (TBARS) (0.705 mg MDA/kg of fish) values of the packaged trout fillets were significantly (*p* ≤ 0.05) observed in CP‐BRPE‐gnAg treatment among the other treatments at the end of the storage period, and CP‐gnAg, CP‐BRPE, and CP treatments were in the next ranks, respectively. Colorimetric analysis of the used films showed that the films containing BRPE depicted color spectra of red to yellow at the same time as the spoilage symptoms initiated in the packaged fillets. It is concluded that BRPE not only increased the preservative effects of chitosan‐polyvinyl alcohol film containing green silver nanoparticles but also can be considered as a natural cost‐effective spoilage indicator of the rainbow trout fillets during cold storage time.

## Graphical abstract

It is concluded that beet root peel extract not only increased the preservative effects of chitosan‐polyvinyl alcohol film containing green silver nanoparticles but also can be considered as a natural cost‐effective spoilage indicator of the rainbow trout fillets during cold storage time.

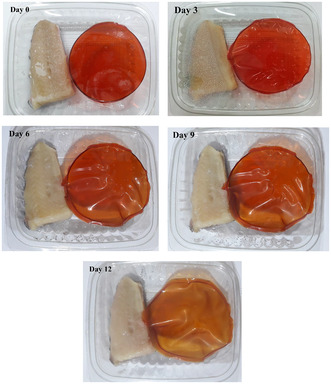

## 1 Introduction

Rainbow trout ( *Oncorhynchus mykiss* ) fish is one of the most abundant and popular edible fish all over the world, which is rich in nutrients such as vitamin D and the best source of omega fatty acids (Bazargani‐Gilani and Pajohi‐Alamoti 2020). The high moisture, free amino acids, unsaturated fatty acids, and pH cause the quality loss and rapid spoilage of fish flesh during the storage period; so, designing an efficient packaging for spoilage monitoring and delay of the fish products can be very helpful in food industries (Bazargani‐Gilani et al. 2021; Ghomi et al. 2021). Considering the consumer demands for natural food products and the environmental pollution issue, biodegradable edible films are widely considered in food packaging.

Chitosan is the de‐acetylated form of chitin that is extracted from the exoskeleton of crustaceans and the cell wall of some fungi, known as a cationic antimicrobial compound that interacts with the bacterial cell surfaces and causes the leakage of cellular nutrients (Yao et al. 2020). Chitosan is used as a unique biopolymer for the preparation of edible packaging due to its non‐toxicity, biocompatibility, biodegradability and film‐forming ability. The chitosan films and coatings are resistant, stable, flexible and transparent and widely used in food packaging by researchers (Shankar, Khodaei, and Lacroix 2021; Yan et al. 2021; Yao et al. 2020). Polyvinyl alcohol is a biodegradable polymer under aerobic and anaerobic conditions. The polyvinyl alcohol as an auxiliary polymer is used in combination with chitosan to improve the film behavior and enhance the strength (Pandey et al. 2020).

Activating the biopolymers with antimicrobial and antioxidant agents can increase their efficiency in the shelf life enhancement of foods during the storage period. Silver nanoparticles can significantly improve the antibacterial activity of the biopolymers. Enrichment of edible films by silver nanoparticles enhances their efficiency in shelf life improvement of the packaged food during storage period (Ahmed et al. 2022; Shankar, Khodaei, and Lacroix 2021). Different chemical and physical methods have been used for the nanoparticles' synthesis. But the physical methods have low efficiency and, as a result, are not economical, and using the chemical techniques leads to the production of toxic byproducts that can seriously threaten the environment (Hassanisaadi et al. 2022). Therefore, using cost‐effective, ecofriendly, efficient, and safe methods is essential for the nanoparticles' synthesis. Recently, using the green techniques in the fabrication of the nanoparticles by chitosan has widely been considered (Madian and Mohamed 2020). Also, smartification of biopolymers can actually inform consumers of the packaged food safety.

Natural indicators are appropriate choices for spoilage monitoring in food packaging. The herbal pigments, such as curcumin, betalains, and shikonin, are the natural, non‐toxic, and water‐soluble compounds that have no aroma and flavor and are responsible for the red, orange, purple, and blue colors of many fruits and flowers with antioxidant, anticancer, and antimicrobial properties (Roy and Rhim 2021). Red beetroot ( *Beta vulgaris* ) peel is rich in red pigments called betalains. Betalains are a group of nitrogenous compounds that are soluble in water, non‐toxic, and sensitive to pH changes. They consist of two parts: yellow‐orange betaxanthin and red‐purple betacyanin (Fu et al. 2020). Previous research demonstrated that the edible films containing beetroot pulp extract displayed a color response followed by the pH changes of the spoiled food (Guo et al. 2021). In addition to the indicatory properties of beetroot pulp extracts, their antioxidant activities have also been shown in several studies that can be correlated to their phenolic compounds (Salamatullah et al. 2021; Šeremet et al. 2020).

Beetroot peel can be considered as a cost‐effective and natural pH indicator in food smart packaging. Since there is no study about the beetroot peel extract as a pH indicator in edible films, we intended to design a chitosan‐polyvinyl alcohol‐based edible film containing green silver nanoparticles (as an antimicrobial agent) and red beetroot peel extract (as an antioxidant agent) in the shelf‐life improvement and spoilage monitoring of the cold‐stored rainbow trout fillet in this study.

## 2 Materials and Methods

### 2.1 Materials

Chitosan (medium molecular weight), polyvinyl alcohol, acetic acid, silver nitrate, methanol, ethanol, 2,2‐diphenyl‐1‐picrylhydrazyl (DPPH), butylated hydroxytoluene (BHT), thiobarbituric acid (TBA), Folin's and Ciocalteu's reagent, sodium carbonate, sodium phosphate, sodium hydroxide, potassium ferricyanide, calcium chloride, chloric acid, gallic acid, trichloroacetic acid, magnesium oxide, boric acid, plate count agar (PCA), violet red bile agar (VRBA), and peptone water were purchased from Merck Company.

### 2.2 Beetroot Peel Extract (BRPE) Preparation

Beetroot peel was provided from the beetroot processing plant. After washing and rinsing, the peels were cut with a knife and dried at room temperature under shade for 10 days. Then, the dried samples were milled and stored at −18°C for next uses. Beet root peel powder was added to each of the water (aqueous extract) and ethanol 70% (ethanolic extract) at a 1:10 ratio. The mixture was shaken at room temperature at 250 rpm for 24 h and then centrifuged (VS4000D, Far Test, Iran) at 1500 rpm for 15 min. Then the supernatant was concentrated in a rotary evaporator (EV311, Lab Tech, Milan, Italy) and stored at −18°C for next analyses (Guo et al. 2021).

### 2.3 Antioxidant Activity of BRPE

#### 2.3.1 DPPH Radical Scavenging Activity and Reducing Power Test

Antioxidant activity of BRPE was measured using DPPH radical scavenging activity (Blois 1958) and reducing power (Oyaizu 1986) tests. BHT solution was used as a positive control in these experiments.

#### 2.3.2 Total Phenolic Content

Total phenolic content of the obtained extracts was determined according to the Singleton, Orthofer, and Lamuela‐Raventós (1999) method using Folin's and Ciocalteu's reagent. The results were reported as mg of gallic acid/g of the extract.

### 2.4 pH‐Sensitivity of BRPE

The pH sensitivity of the BRPE was measured by UV–Visible spectroscopy in the wavelength range of 300–900 nm. 1 mL of the BRPE was mixed with 1 mL of distilled water, and then the pH of the solutions was adjusted from 3 to 12 with a pH meter (E520, Metrohm Herisau, Bern, Switzerland) using HCl and NaOH (0.1 M), respectively. Then, the absorbance of the obtained solutions was read at the wavelengths of 300–900 nm using a spectrophotometer (Thermo Spectronic, UK) (Guo et al. 2021).

### 2.5 Green Synthesis of Silver Nanoparticles Using Chitosan

100 mL of yellow chitosan solution (6%) was mixed to 0.5 mL of the silver nitrate solution (0.001 M) and continuously mixed at 90°C for 6 h to obtain a brown color (Pandey et al. 2020).

### 2.6 UV–Visible Spectroscopy of nAg

In order to prove the reduction of silver nitrate solution by chitosan to silver nanoparticles, the absorbance of chitosan solution containing silver nanoparticles was read by UV–Visible spectrophotometer (Thermo Spectronic, UK) at a wavelength of 300–600 nm.

### 2.7 Optical Microscope and Scanning Electron Microscope (SEM) Images of nAg

Images of the biosynthesized silver nanoparticles were provided by an optical microscope (Olympus, CX31, Japan) and SEM (Jeol JSM‐840, Japan) at magnifications of 1000× and 10,000×, respectively.

### 2.8 Preparation of the Films

Chitosan (6%) and PVA (12%) solutions were prepared in acetic acid (2%) solvent. Then, the mixture of PVA‐Chitosan was prepared in a ratio of 7:3 in CP film (chitosan‐polyvinyl alcohol film). For the films containing nAg, the obtained chitosan‐nanosilver solution in the previous section (2.5. Green synthesis of silver nanoparticles using chitosan) was used (CP‐gnAg film (chitosan‐polyvinyl alcohol‐green nanosilver particles film)). Next, 3 mL of BRPE and glycerol (30% of the total solids weight of the films) were added to the CP solutions as the plasticizer and stirred continuously for CP‐BRPE film (chitosan‐polyvinyl alcohol‐beetroot peel extract film), and finally 3 mL of BRPE and glycerol (30% of the total solids weight of the films) were added to the chitosan‐nanosilver solution for CP‐BRPE‐gnAg film (chitosan‐polyvinyl alcohol‐beet root peel extract‐green nanosilver particles film). The obtained solution was poured into petri dish plates with a diameter of 8 cm and dried on a flat surface for 3 days. After drying the films, they slowly peeled off the plate (Pandey et al. 2020).

### 2.9 Physicochemical Analysis of the Films

#### 2.9.1 Film Thickness

The thickness of each film was measured using a digital micrometer (IP65 Alpa Exacto, Milan, Italy) with an accuracy of 0.01 mm from 5 different points of the films and reported as the mean value (Yan et al. 2021).

#### 2.9.2 Mechanical Analysis of the Films

The mechanical features of the films were determined according to the ASTM D882 standard. 1 × 6 cm pieces of the films were placed between the two jaws of the texture analyzer (Z 2.5, Zwick, Instron, Germany) and stretched at a speed of 50 mm/min. Then, the tensile strength (TS), the percentage of elongation at break (EB), and the Young's modulus (YM) were measured (Koosha and Hamedi 2019; ASTM 2012).

#### 2.9.3 Water Vapor Permeability (WVP)

The water vapor permeability (WVP) of the films was measured according to the ASTM D882‐12 standard method. Five replications were considered for the films and their mean values were calculated.

#### 2.9.4 X‐Ray Diffraction (XRD)

An X‐ray diffraction (XRD) system was used to determine the crystallinity degree of the produced films. The test was recorded in the range of 10°–80° and at the speed of 0.5° per second (Ali and Ahmed 2021).

#### 2.9.5 pH‐Sensitivity of CP and CP‐BRPE Films

The pieces (10 × 10 mm) of the CP and CP‐BRPE composite films were immersed for 5 min in the acidic to alkaline solutions in the pH range of 3–12 using HCl or NaOH (0.1 M) (Yan et al. 2021).

### 2.10 Preparation of Treatments

Fresh rainbow trout fish were provided from a fish processing plant in Hamedan, Iran. They were transferred to the laboratory in the insulated containers containing ice packs. After washing and cutting the fillets, the 10 g pieces of the fish fillet were placed between two films from each treatment, and the films were closed by a thermal sewing machine (PFS‐200, Iran Tarazo, Tehran, Iran). Each package was kept in a refrigerator at 4°C and analyzed for the microbial, chemical, and sensory features on 0, 3, 6, 9, and 12 days of the storage period. The studied treatments were as follows: (1) C (control, fillets without packaging), (2) CP (fillets packaged with chitosan‐polyvinyl alcohol film), (3) CP‐gnAg (fillets packaged with chitosan‐polyvinyl alcohol‐green nanosilver particles film), (4) CP‐BRPE (fillets packaged with chitosan‐polyvinyl alcohol‐beetroot peel extract film), (5) CP‐BRPE‐gnAg (fillets packaged with chitosan‐polyvinyl alcohol‐beet root peel extract‐green nanosilver particles film).

### 2.11 Microbial Analysis

To measure the microbial population, 10 g of the fish fillet was mixed with 90 mL of peptone water (0.1%) in a stomacher bag and stomached at 250 rpm for 60 s. After preparing the serial dilutions, 0.1 mL of the samples were cultured in PCA culture medium and incubated at 37°C for 24 h for a total viable count. Psychrotrophic bacteria were detected in PCA culture medium at 7°C for 10 days. A double‐layer culture form was used for *Enterobacteriaceae* count in VRBA culture medium at 37°C for 24 h. Microbiological evaluation was expressed as the log of the number of colony‐forming units (CFU/g) (Yousef and Carlstrom 2003).

### 2.12 Chemical Analysis

#### 2.12.1 pH Measurement

10 g of the fish sample was mixed with 50 mL of distilled water. After homogenization, the pH of the samples was measured using a pH meter (E520, Metrohm Herisau, Bern, Switzerland) (Brannan 2008).

#### 2.12.2 Thiobarbituric Acid Reactive Substances (TBARS)

10 g of the fish fillet was homogenized with 1 mL of BHT (1 mg/mL) and 35 mL of trichloroacetic acid (5%) for 1 min. Then, the sample was filtered with Whatman No. 1 paper and adjusted to 50 mL with trichloroacetic acid (5%). 5 mL of this solution was mixed with 5 mL of TBA solution (0.02 M) and placed in the boiling water bath for 60 min. After cooling the solutions, the absorbance was read using a spectrophotometer (Thermo Spectronic, UK) at a wavelength of 532 nm (Pikul, Leszczynski, and Kummerow 1989).

#### 2.12.3 Total Volatile Basic Nitrogen (TVB‐N)

In order to measure the amount of TVBN, 10 g of the fish sample was mixed with 2 g of the magnesium oxide and 300 mL of the distilled water and some glass pearls in the distillation flask of the Kjeldahl set. After heating the flask, the resulted vapors were trapped in the 25 mL of boric acid (2%) containing the reagent. This process continued until the boric acid (red) discolored to green and then titrated using sulfuric acid (0.1 N) until the appearance of a stable red color (Fernández, Aspe, and Roeckel 2009). The TVB‐N value was calculated from the following formula:

$$\mathrm{TVB}-N\;\left(\mathrm{mg}/100\;g\right)=\text{Volume of the consumed acid}\times 14$$

### 2.13 pH Indicator Property of the Films

To measure the pH indicator property of the films, 100 g of the fish fillet was placed in sterile containers. The CP‐BRPE films were glued under the lid of the containers using heat, and then the samples were stored in the refrigerator at 4°C ± 1°C. By increasing the storage time, film discoloring was measured by three factors of L*, a*, and b* using a colorimeter (410‐Minolta CR model, Japan). L* is the transparency index of the sample (black = 0 and white = 100), a* is the redness index (green = −60 and red = +60), and b* is the yellowness index (blue = −60 and yellowness = +60) (Guo et al. 2021).

### 2.14 Sensory Analysis

20 (10 females and 10 males, 20–30 years old) trained students of the Food Hygiene and Quality Control Department were invited to participate in the sensory analysis. Sensory characteristics were determined using a 5‐point hedonics scale (1–5). The panelists were not informed about the experimental approach, and the samples were blind‐coded with 3‐digit random numbers. Fresh fish fillet was concerned as the reference. The panelists assessed the color, odor, and overall acceptability of the fillets. In case of reaching the scores of the sensory property below 3.0, fish fillets were rejected. The factors of odor, color, and overall acceptability were evaluated and graded from 1 to 5. (1: Very poor, 2: Unacceptable or poor, 3: Acceptable or average, 4: Satisfactory or good, 5: Very satisfactory or very good).

### 2.15 Statistical Analysis

All tests were performed in triplicate. The obtained data were illustrated as mean values ± standard deviations (SD). Variance analysis (ANOVA) with the Tukey test was used at the significance set of *p* ≤ 0.05 to compare differences among the studied groups using SPSS software (IBM SPSS statistics 21).

## 3 Results and Discussion

### 3.1 Antioxidant Activity and Total Phenolic Content of BRPE

The results of the antioxidant activity tests and total phenolic content of the studied extracts are shown in Table 1. According to the obtained findings, ethanolic BRPE significantly (*p* ≤ 0.05) exhibited higher antioxidant activity (DPPH radical scavenging activity (43.02%) and reducing power (2.87)) and total phenolic content (360.50 mg GAE/g) than aqueous extract. Therefore, ethanol 70% solvent is an appropriate safe solvent in the extraction of the effective ingredients of BRPE and was chosen for the film fabrication in this study. Šeremet et al. (2020) used several extraction methods (maceration, microwave, and ultrasound) for producing the BRPE using water solvent. They reported that the maceration technique was the most effective method in releasing the phenolic compounds, followed by the strong antioxidant activity of the obtained BRPE among the other methods. In another study, the total phenolic content of aqueous‐methanolic (50%) and water BRPE was 24.04 and 14.89 mg GAE/g, respectively (Salamatullah et al. 2021). Consistent with our study, they concluded that the aqueous‐alcoholic solvent showed more ability in releasing the phenolic ingredients of beet root peel than the water solvent.

**TABLE 1:** DPPH radical scavenging activity (%), reducing power, and total phenolic content of beet root peel extracts.

Extracts	RSA%	Reducing power	Total phenolic content (mg GAE/g)
Aqueous BRPE	40.67 ± 1.5^b^	0.11 ± 0.02^c^	276.44^b^
Ethanolic BRPE	43.02 ± 1.1^c^	2.87 ± 0.002^b^	360.50^a^
BHT	92.66 ± 0.9^a^	3.04 ± 0.01^a^	—

*Note:* Different letters indicate a statistically significant difference (*p* ≤ 0.05) for the same column.

### 3.2 pH Sensitivity of BRPE

**FIGURE 1:**
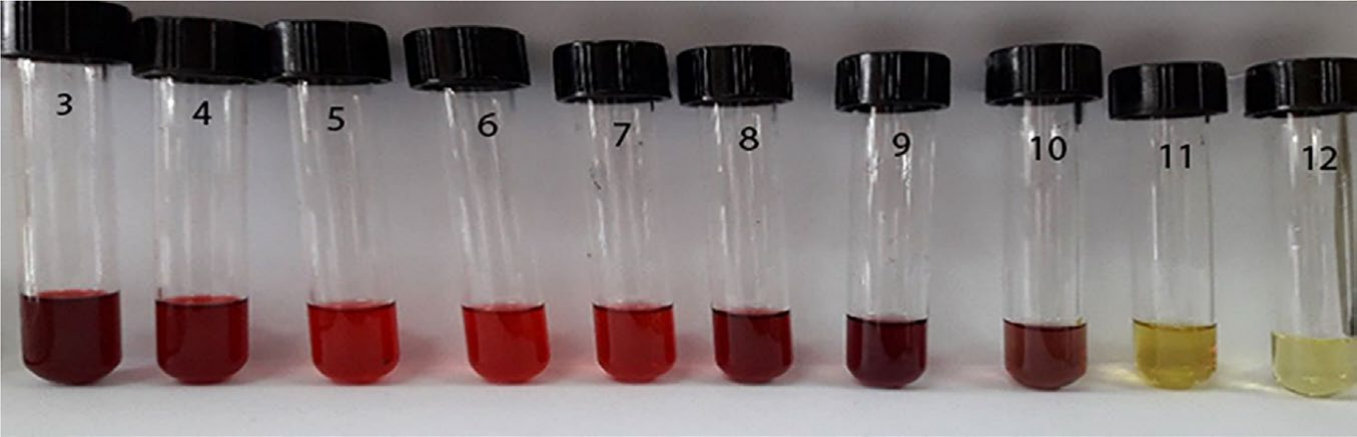
The color response of BRPE against different pHs (3–12).

Discoloration of BRPE under pH changes is depicted in Figure 1. According to the obtained results, this extract displayed the colors of deep red at pH 3, dark red at pH 4, bright red at pH 5–7, purple at pH 8, dark brown at pH 9, brown at pH 10, yellow at pH 11, and light yellow at pH 12. Guo et al. (2021) reported that the color of the beet root extract changed from bright red to yellow in the pH range of 3–10. They concluded that the reversible transformation of anthocyanin compounds against pH changes is responsible for this phenomenon.

### 3.3 UV–Visible Spectroscopy of BRPE

**FIGURE 2:**
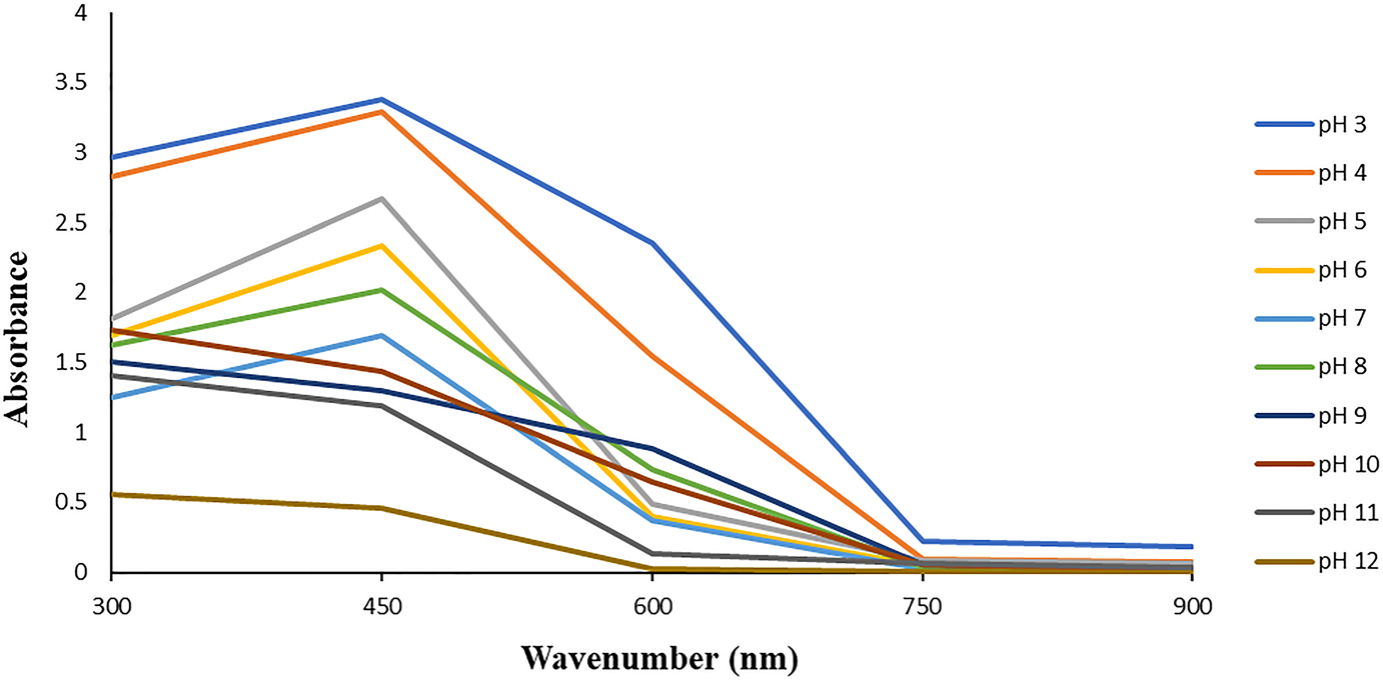
UV–Visible spectra of BRPE in wavenumber range of 300–900 nm.

Figure 2 illustrates the UV–Visible spectra of the discolored extracts in different pHs. According to the UV–Visible spectra, each of the discolored extracts in different pHs created one spectrum in certain wavelengths (Figure 2) so that the highest and lowest spectra were revealed in 450 nm that were correlated to pH 3 and 12, respectively. On the other hand, by increasing the pH values, the absorbance intensity of the samples relatively decreased. It seems that the higher pH destroys the color intensity of the BRPE. But previous researchers have found that the beet root extract exhibited the highest absorbance in pH 3 and 4 at 409 nm. Furthermore, they reported that by increasing the pH values, the absorbance intensity of the beet root extract increased. These contradictions can be related to the different nature of the beet root and beet root peel extracts (Chhikara et al. 2019; Guo et al. 2021).

### 3.4 Biosynthesis of nAg

#### 3.4.1 UV–Visible Spectroscopy of nAg

**FIGURE 3:**
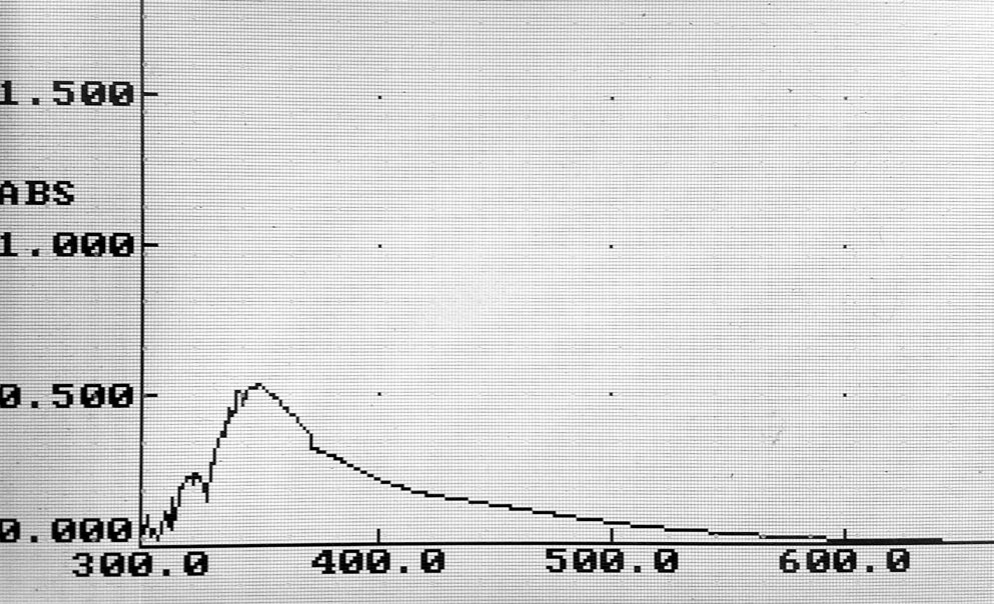
UV–Visible spectra of the biofabricated nAg.

The color change of chitosan solution from light yellow to brown indicated the silver nitrate reduction and nanosilver production, which was also confirmed by UV–Visible spectroscopy. According to the obtained findings, the biofabricated nAg revealed a peak at the wavelength of 351 nm (Figure 3). Pandey et al. (2020) reported the peak of the biosynthesized silver nanoparticles in the range of 427 nm. Shankar, Khodaei, and Lacroix (2021) observed this peak in the range of 420 nm, and Selvaraj, Thangam, and Fathima (2018) obtained it in 400 nm. These differences can be related to the different physicochemical features (such as size, zeta‐potential, and polydispersity index) of the produced silver nanoparticles.

#### 3.4.2 Optical Microscope and Scanning Electron Microscope (SEM) Images

**FIGURE 4:**
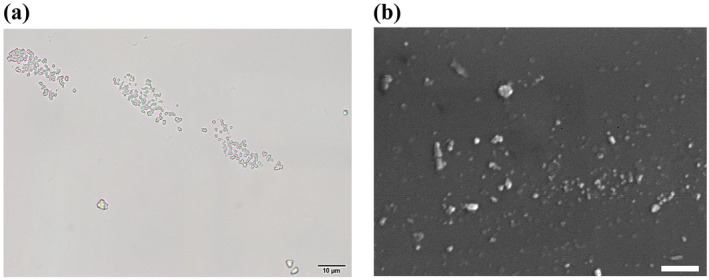
The optical microscope (a) and SEM (b) images of the biofabricated nAg in magnifications of 1000× and 10,000×, respectively.

Figure 4a represents the optical microscope image of the biosynthesized nAg colloids in the magnification of 1000× and scale of 10 μm. Spherical particles with a blue‐green metallic luster confirmed the biofabrication of silver nanoparticles. Figure 4b depicts an SEM image of the biosynthesized nAg in the magnification of 10,000× and scale of 1 μm. According to the obtained images, the obvious separate spherical particles with a diameter of 51 nm prove bio‐fabrication of nAg using silver nitrate reduction by chitosan successively. Pandey et al. (2020) reported that the diameter of biosynthesized silver nanoparticles using chitosan was in the range of 70–130 nm. In another study, the diameter of the bio‐fabricated silver nanoparticles was obtained by TEM images in the range of 1–40 nm (Kumar et al. 2021). Hassanisaadi et al. (2022) biofabricated the spherical silver nanoparticles with a diameter of 40.99–57.42 nm using *Aloysia citrodora* leaf extract.

### 3.5 Physicochemical Characteristics of the Films

#### 3.5.1 Film Thickness

Table 2 represents the physicochemical properties (thickness, TS, EB, and YM) of the studied films. CP film had the lowest thickness (0.19 mm) among the films. Adding nAg or BRPE to the films increased the film thickness to 0.23 mm. The results of Gasti et al. (2021) showed that the addition of *Phyllanthus reticulatus* extract to the matrix of chitosan‐methylcellulose film increased the film thickness due to the soluble solid enhancement of it. Medeiros Silva et al. (2020) reported that adding anthocyanins to the starch‐based films increased their thickness. According to the findings of Pandey et al. (2020) and Shah, Hussain, and Murtaza (2018), incorporating silver nanoparticles into chitosan‐based edible films increased their thickness.

**TABLE 2:** Physicochemical characteristics of the designed films.

Films	Thickness (mm)	TS (MPa)	EB (%)	YM (MPa)
CP	0.19 ± 0.02^b^	3.20 ± 0.04^d^	71.23 ± 0.17^d^	4.55 ± 0.03^a^
CP‐nAg	0.23 ± 0.02^a^	3.40 ± 0.08^c^	90.63 ± 0.13^c^	4.46 ± 0.01^b^
CP‐BRPE	0.23 ± 0.01^a^	4.20 ± 0.05^a^	118.18 ± 0.45^a^	3.04 ± 0.02^d^
CP‐BRPE‐nAg	0.23 ± 0.02^a^	3.70 ± 0.06^b^	96.08 ± 0.31^b^	3.80 ± 0.01^c^

*Note:* Different letters indicate a statistically significant difference (*p* ≤ 0.05) for the same column.

#### 3.5.2 Mechanical Features of the Films

TS, EB, and YM are three important characteristics in food packaging (Gasti et al. 2021). According to Table 2, the CP film had the highest YM (4.55 MPa) and the lowest TS (3.20 MPa) and EB (71.23%). Addition of BRPE and gnAg to the CP film significantly (*p* ≤ 0.05) decreased the YM and increased TS and EB so that the highest TS (4.20 MPa) and EB (118%) belonged to the CP‐BRPE film, which is due to the bound formation between anthocyanin ingredients of BRPE and CP matrix (Chen et al. 2021; Yan et al. 2021). Therefore, CP‐BRPE film was smoother and more flexible than the others. It seems that adding nAg to the treatment containing BRPE in CP‐BRPE‐gnAg film led to a decrease in the TS (3.70 MPa) and EB (96.08%) as compared to the CP‐BRPE film that can be correlated to a decrease in the film matrix cohesion due to the silver nanoparticles presence (Pandey et al. 2020). Gasti et al. (2021) reported that adding *Phyllanthus reticulatus* extract to the chitosan‐methylcellulose matrix caused a decrease in the EB and an increase in TS and YM. This can be related to the nature of the bonds between phenolic compounds and film materials, which leads to a decrease in the film flexibility. Yao et al. (2020) found that adding cactus pears betalains to the ammonium chitosan‐polyvinyl alcohol matrix caused an increase in EB in the studied films. In another study, adding *Eriobotrya japonica* leaves' extract to the starch‐banana peel film increased EB and decreased TS and YM of them (Medeiros Silva et al. 2020). Koosha and Hamedi (2019) reported that following the addition of black carrot extract to chitosan‐polyvinyl alcohol film, TS increased. This can be related to the hydrogen bonding between the OH^+^ groups of anthocyanins and chitosan‐polyvinyl alcohol film.

#### 3.5.3 Water Vapor Permeability (WVP)

**FIGURE 5:**
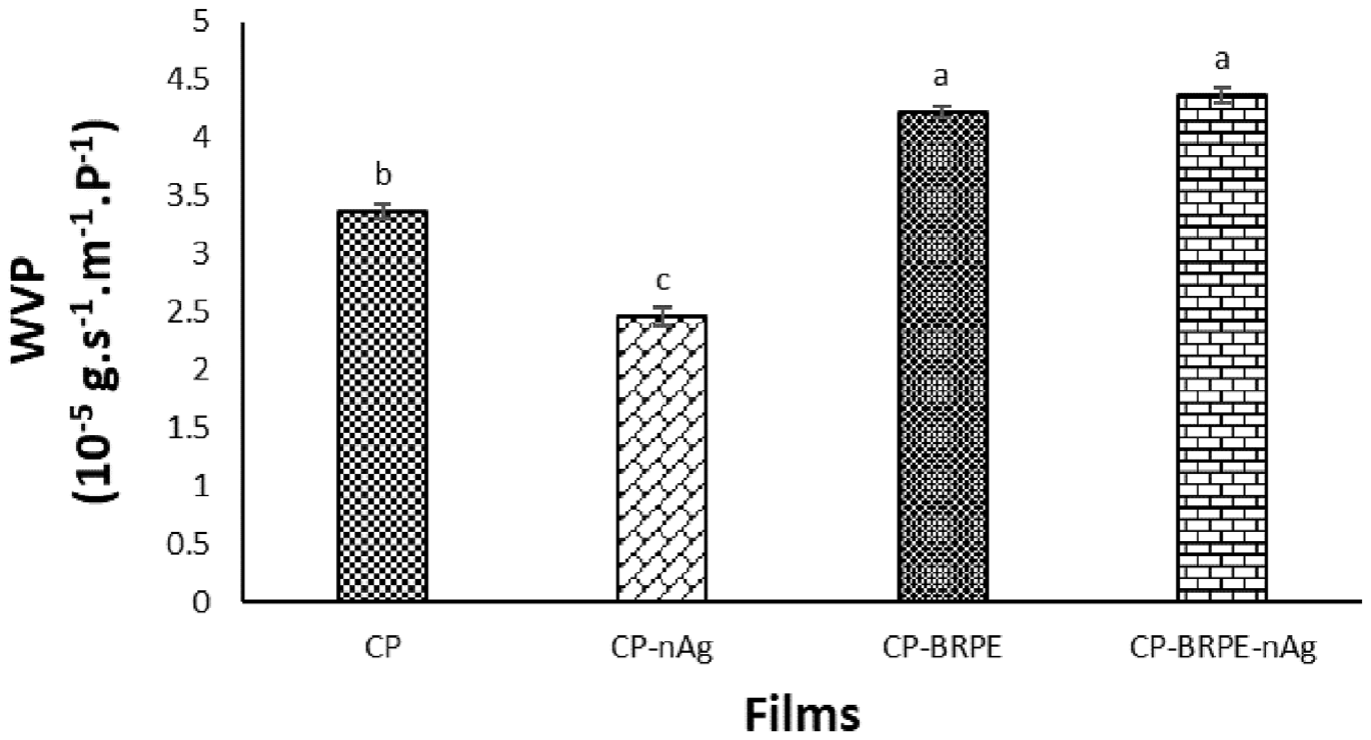
The water vapor permeability of the designed films. Films: CP (Chitosan‐polyvinyl alcohol), CP‐nAg (Chitosan‐polyvinyl alcohol‐nano silver particles), CP‐BRPE (Chitosan‐polyvinyl alcohol‐beetroot peel extract), and CP‐BRPE‐nAg (Chitosan‐polyvinyl alcohol‐beet root peel extract‐nano silver particles). Different letters indicate a statistically significant difference (*p* ≤ 0.05).

Edible films act as a barrier between the food and the environment and prevent the transfer of gases and water vapor to the food. Therefore, WVP is a determining factor in evaluating the film efficiency (Yong, Liu, et al. 2019; Yong, Wang, et al. 2019). Figure 5 illustrates the WVP of the studied films. The CP film had a WVP of 3.36 10^−5^ g/s/m/P that can be related to the hydrophilic polyvinyl alcohol presence. The lowest WVP was related to the CP‐nAg film with a rate of 2.45 10^−5^ g/s/m/P. The presence of silver nanoparticles acts as a barrier against water vapor due to the filling of empty spaces of matrix and causes a decrease in permeability, which was confirmed in the CP‐nAg film. The BRPE significantly (*p* ≤ 0.05) increased the WVP of the films, so that in the CP‐BRPE‐nAg film, the property of the BRPE has overcome the presence of silver nanoparticles and the highest WVP with a value of 4.37 10^−5^ g/s/m/P belonged to it. Yong, Liu, et al. (2019) showed that the presence of low percentages of black and purple rice extract in chitosan films caused a decrease in WVP; but, the higher concentrations of the extract created more free spaces in the film matrix that caused WVP to increase. Koosha and Hamedi (2019) showed that the anthocyanins in the edible films increased WVP, which was consistent with our results. But, according to the findings of Gasti et al. (2021), the polyphenols and flavonoids of *Phyllanthus reticulatus* extract decreased the film WVP, which can be probably related to the hydrogen bond formation between extract and chitosan/methyl cellulose film. Guo et al. (2021) observed that the presence of red beetroot extract in the watermelon pectin‐based edible film caused a decrease in WVP in the studied films. It has been reported that water vapor transmission probably occurs through the amorphous regions of the films, so the crystallinity degree of the films can affect the WVP (Yong, Liu, et al. 2019). The higher the crystallinity of the film, the lower the amorphousness degree. According to the XRD results (Figure 5), the lowest crystallinity degree belongs to the CP‐BRPE film, which caused the highest WVP rate of it among the other films.

#### 3.5.4 pH‐Sensitivity of CP and CP‐BRPE Films

**FIGURE 6:**
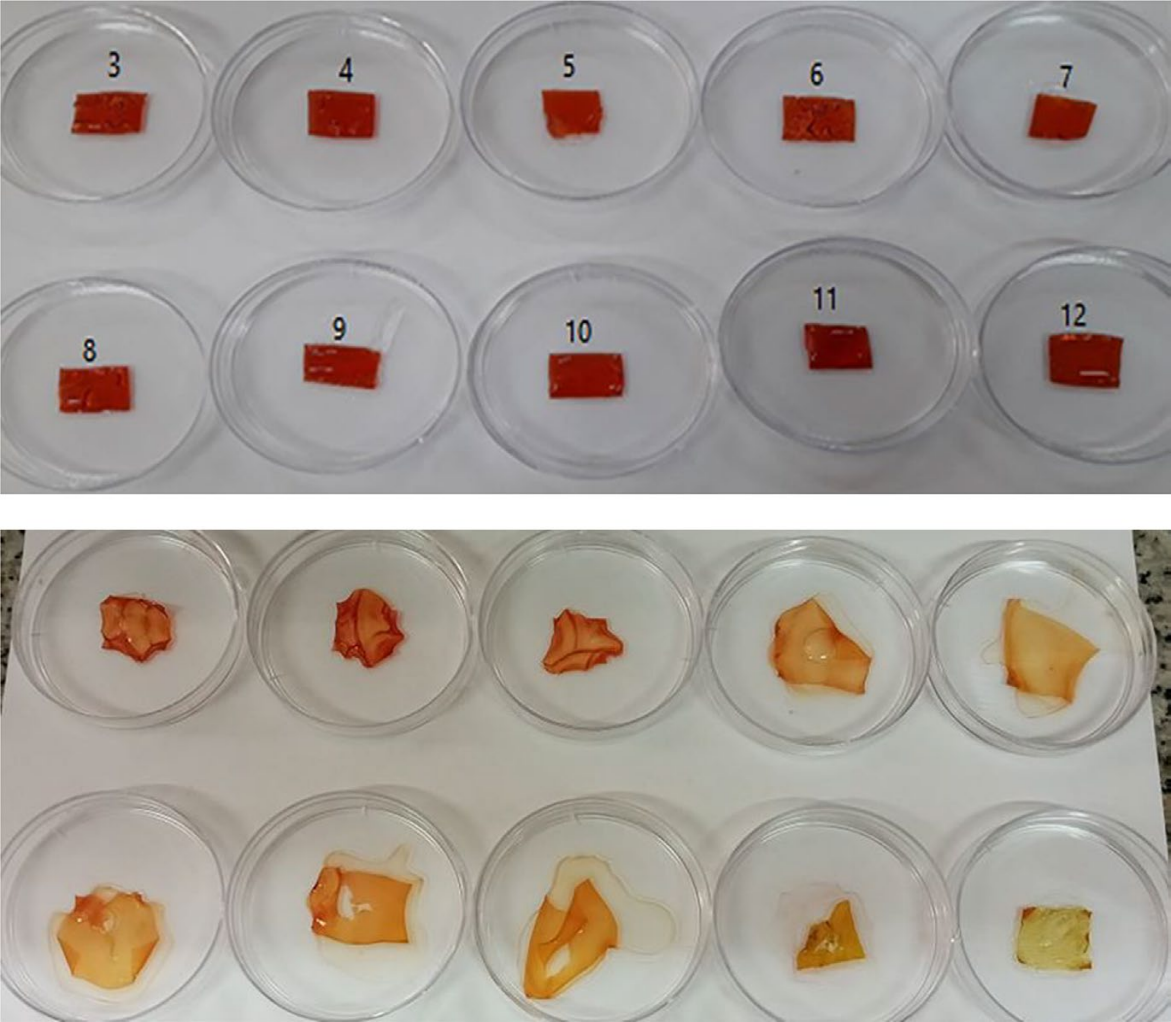
Color response of the CP‐BRPE film against different pHs (3–12).

Figure 6 depicts the color changes of the CP‐BRPE film against different pHs. Since no color change was observed in the CP film against a pH range of 3–12, its images were not shown. The color of CP‐BRPE film changed from red in the pH range of 3–5 to light brick red in the pH range of 6–9, orange at pH 10, brown at pH 11, and yellow at pH 12. The color changes of the films were not fully consistent with the BRPE, which could be due to the hydrogen combination of the BRPE with chitosan, which affected the color response of the anthocyanin and betalain compounds. The color changes of the films containing BRPE at different pHs are due to the betalain ingredients. Betalains are the red beetroot peel pigments that dissolve in water and are composed of betacyanins (prebetanin, betanin, neo‐betanin, and isobetanin) and betaxantins. These compounds are very sensitive to pH and change with different pHs (Chhikara et al. 2019; Fu et al. 2020). Guo et al. (2021) observed the color changes of beet root extract from acidic to alkaline pH from cherry red (pH = 3–4) to bright red (pH = 5–6), orange (pH = 7–8), brown (pH = 9), and yellow (pH = 10) respectively.

#### 3.5.5 X‐Ray Diffraction (XRD) Analysis

**FIGURE 7:**
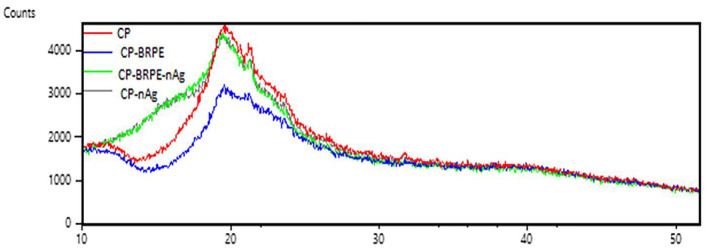
XRD spectra of the designed films. Films: CP (Chitosan‐polyvinyl alcohol), CP‐nAg (Chitosan‐polyvinyl alcohol‐nano silver particles), CP‐BRPE (Chitosan‐polyvinyl alcohol‐ beetroot peel extract), and CP‐BRPE‐nAg (Chitosan‐polyvinyl alcohol‐beet root peel extract‐nano silver particles).

Figure 7 represents the X‐ray diffraction (XRD) analysis of the studied films. According to the obtained results, all films created peaks at 19.4°, 21.3°, and 23.6° of 2*θ* with different intensities. The CP‐nAg film also showed a small peak at 37° that is related to the silver nanoparticles. The CP‐BRPE‐nAg film did not show this small peak, which is probably due to the amorphous non‐crystalline structure of the BRPE, which was proved by the lowest absorption intensity in the peaks of the CP‐BRPE film. Pandey et al. (2020) found the peaks at 19.4° and 22.3° for chitosan‐polyvinyl alcohol film and an extra peak for chitosan‐polyvinyl alcohol film containing green silver nanoparticles at 38° of 2*θ*. Ali and Ahmed (2021) reported two large and small peaks for chitosan‐polyvinyl alcohol films at 19.3° and 39.6° of 2*θ*, respectively. Koosha and Hamedi (2019) reported that the obtained peaks of chitosan‐polyvinyl alcohol film containing bentonite and black carrot extract were similar to the free film peaks and concluded that anthocyanins have no effect on the crystalline nature of the chitosan‐polyvinyl alcohol films and only decrease the peak intensities. Yong, Liu, et al. (2019) showed that the amorphous structure of black and purple rice extracts caused a decrease in the peak intensity of the chitosan‐based film due to the hydrogen bond formation between the extracts and chitosan that led to the interference in the semi‐crystalline nature of chitosan. In another study, by increasing the purple potato extract percentage in chitosan film, the obtained peak intensity was lower than the free films (Yong, Wang, et al. 2019). Generally, by increasing the crystallinity degree of the films, they become more fragile and the EB values of them decrease. Therefore, the film that shows the lowest absorption in the XRD analysis has the highest EB value. In other words, there is a reverse relation between EB and XRD values in the films (Yong, Liu, et al. 2019; Yong, Wang, et al. 2019). Therefore, our finding accuracy is confirmed by the lowest absorbance in the XRD analysis and the highest EB value (118.18%) in the CP‐BRPE film.

### 3.6 Microbial Analysis of the Packaged Fillets

**FIGURE 8:**
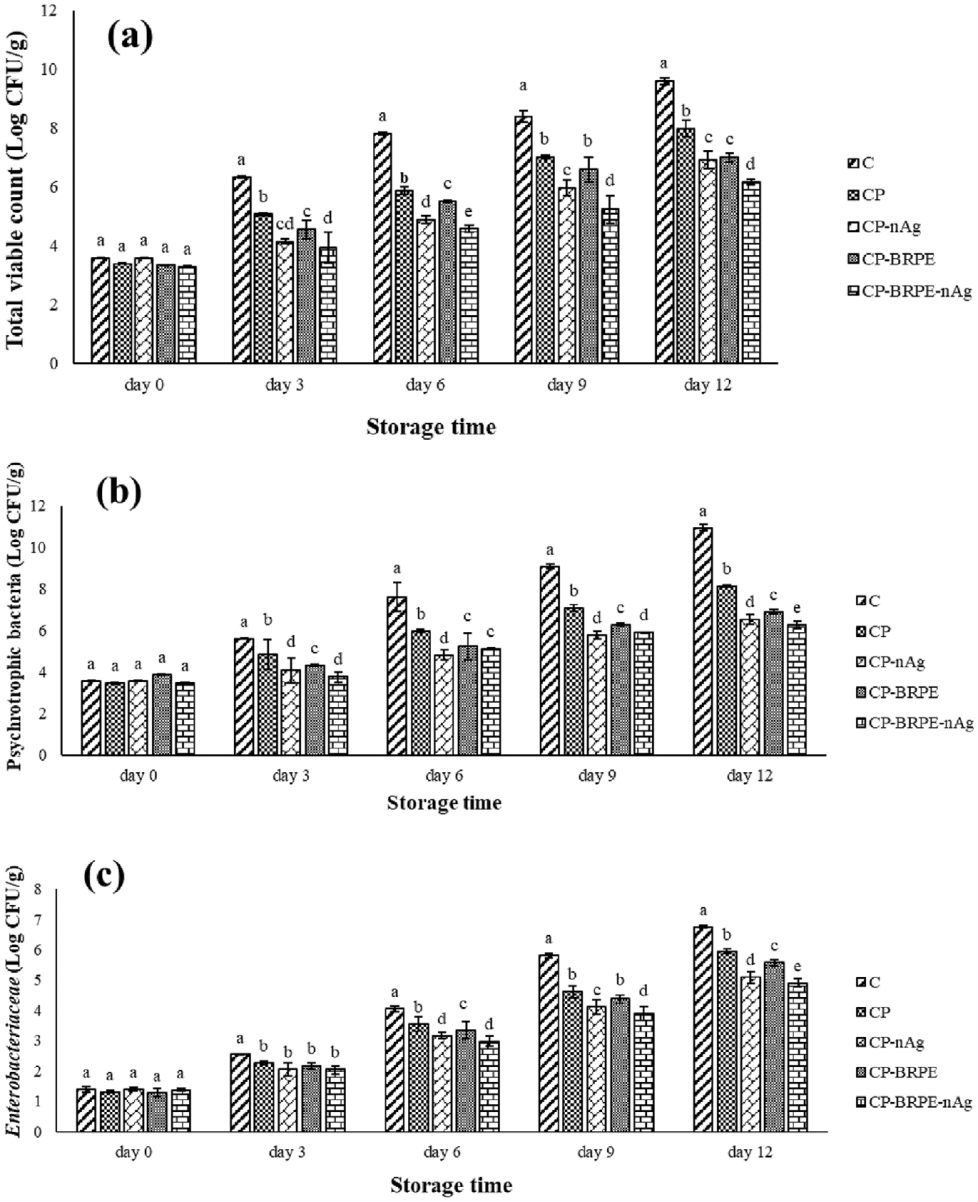
(a–c) The changes in the microbial population (a: total viable count, b: psychrotrophic bacteria, c: Enterobacteriaceae) of the studied treatments during storage period. Treatments: C (control, fillets without packaging), CP (fillets packaged with chitosan‐polyvinyl alcohol), CP‐nAg (fillets packaged with chitosan‐polyvinyl alcohol‐nano silver particles), CP‐BRPE (fillets packaged with chitosan‐polyvinyl alcohol‐beetroot peel extract), and CP‐BRPE‐nAg (fillets packaged with chitosan‐polyvinyl alcohol‐beet root peel extract‐nano silver particles). Different letters indicate a statistically significant difference (*p* ≤ 0.05) for the same day.

Seafood spoilage mainly depends on the bacterial population, which determines the consuming possibility of the product for the consumers (Wu et al. 2019). Figure 8a–c depicts the microbial analysis (total viable count (a), psychrotrophic bacteria (b), and *Enterobacteriaceae* (c)) of the packaged trout fillets during the storage period. The acceptable limit of the bacteria for the consumption of food is < 7 log CFU/g sample (ICMSF 1986). The control group significantly (*p* ≤ 0.05) showed the highest growth rate compared to the others and spoiled after the 3rd day. The lowest total viable count (6.17 log CFU/g), psychrotrophic bacteria (6.27 log CFU/g), and *Enterobacteriaceae* (4.9 log CFU/g) were significantly (*p* ≤ 0.05) observed in CP‐BRPE‐gnAg treatment among the other treatments at the end of the storage period, and CP‐gnAg, CP‐BRPE, and CP treatments were in the next ranks, respectively. According to the obtained results, gnAg and BRPE exhibited the antibacterial activities in the packaged fish fillets. Yavuzer, Özogul, and Özogul (2020) found that the icing with red beetroot extract improved the shelf life of the rainbow trout and created good quality features, which is due to the antimicrobial properties of the red beetroot extract. They reported that the red beet root extract is rich in phenolic acids, especially chlorogenic, gallic, protocatechuic, and caffeic acid, with antimicrobial activities and health benefits. The antibacterial effects of the red beetroot ethanolic extract against *Bacillus*, *Staphylococcus*, and *Escherichia coli* bacteria were proved by Nwankwo (2021). They found the various phytochemical ingredients in beetroot extract, such as saponin, tannins, alkaloids, flavonoids, and cardiac glycosides. Maqbool et al. (2021) observed that the shelf life of the BRPE‐coated mahseer steaks elongated for more than 6 months during the frozen storage. They believed that the high concentrations of betalains, as the phenolic compounds of BRPE, possessed strong antioxidant and antimicrobial activities against foodborne pathogens. According to their reports, the beetroot peel is composed of 50% of phenolic compounds, but the beetroot flesh consists of only 13%. Fadıloğlu and Emir Çoban (2018) found that the positive effect of chitosan‐based edible film containing sumac extract on the shelf life improvement of the cold‐stored salmon fillet was related to inhibiting the microbial growth, followed by preventing fish spoilage.

Dissolution of the silver nanoparticles causes the release of the silver ions, which have impressive antimicrobial activity. The silver ions increase the bacterial membrane permeability that leads to the disturbance in the transportation of the vital molecules in the cells, followed by the block of the respiratory enzymes, essential proteins, nucleic acid replications (DNA and RNA), and finally cell death (Kailasa et al. 2019; Mohamed and Madian 2020).

Pandey et al. (2020) reported that the antibacterial effects of chitosan film are related to the disturbance created in the permeability of the bacterial cell membrane due to the positive and negative charges of the chitosan and bacterial membrane, respectively, that was followed by electrostatic interaction between them. These phenomena can lead to the osmotic pressure disruption and bacterial cell wall hydrolysis.

### 3.7 Chemical Analysis of the Packaged Fillets

#### 3.7.1 pH Value

**FIGURE 9:**
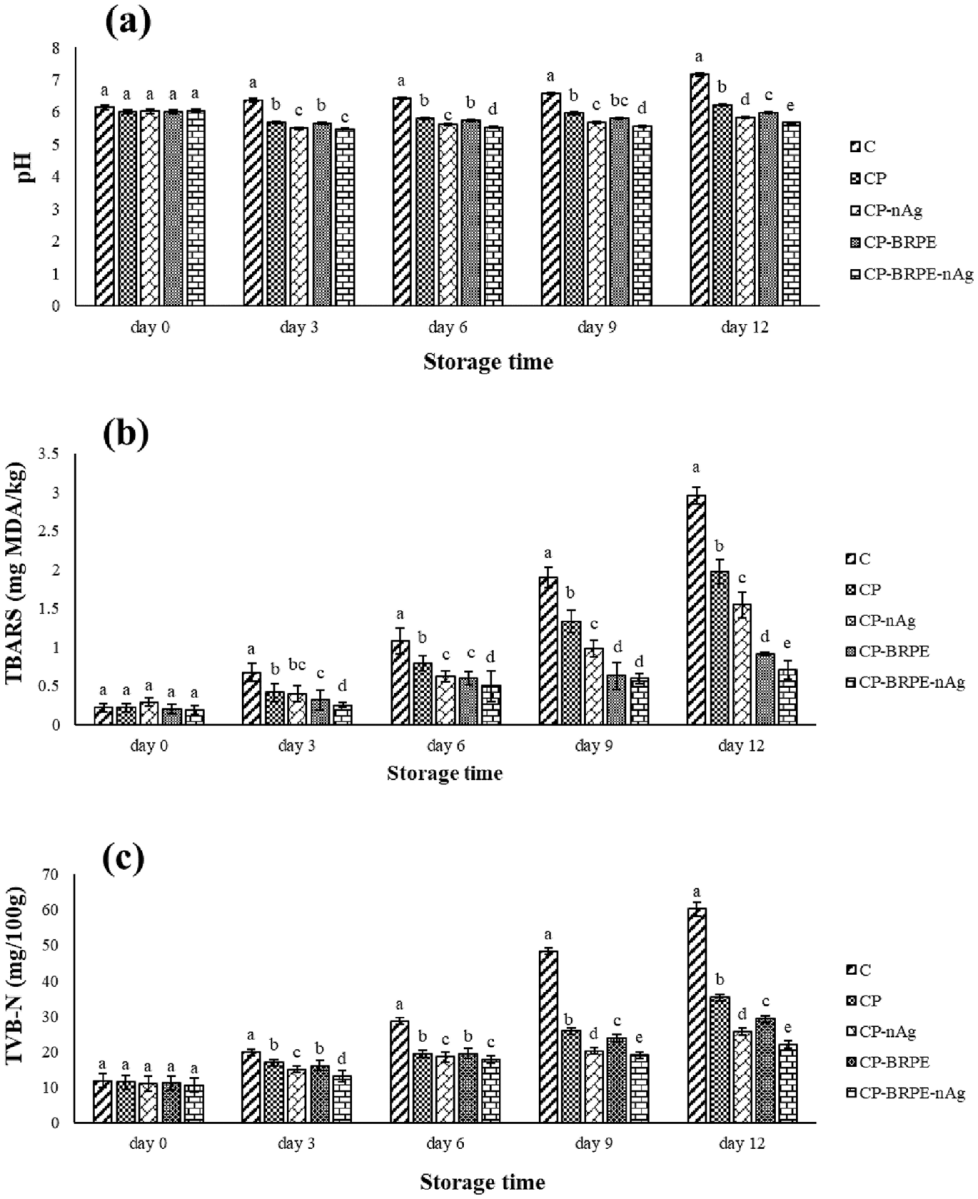
(a–c) The changes in the chemical features (a: PH, b: TBARS, and c: TVB‐N) of the studied treatments during storage period. Treatments: C (control, fillets without packaging), CP (fillets packaged with chitosan‐polyvinyl alcohol), CP‐nAg (fillets packaged with chitosan‐polyvinyl alcohol‐nano silver particles), CP‐BRPE (fillets packaged with chitosan‐polyvinyl alcohol‐beetroot peel extract), and CP‐BRPE‐nAg (fillets packaged with chitosan‐polyvinyl alcohol‐beet root peel extract‐nano silver particles). Different letters indicate a statistically significant difference (*p* ≤ 0.05) for the same day.

Figure 9 depicts the pH values of the packaged fillets during 12 days of the storage period. Due to the microbial growth and decomposition of protein substances in the fish fillets, their pH values increased, which was clearly observed in the control group, so that the pH value of it increased from 6.14 on the first day until 7.17 on the last day. While this range was 6.45–5.66, 6.02–5.82, 6.00–5.97, and 6.01–6.22 in the CP‐BRPE‐gnAg, CP‐gnAg, CP‐BRPE, and CP groups, respectively. Similar to the microbial findings, the lowest pH of the studied fillets was observed in CP‐BRPE‐gnAg treatment during the storage time, and the CP‐gnAg, CP‐BRPE, and CP groups were in the next ranks, respectively. It seems that the designed edible films could prevent the pH value increase of the fillets by decreasing their microbial population during the cold storage period. The presence of betalanin and silver nanoparticles in the films decreased the microbial growth of the fillets, followed by inhibiting the decomposing enzyme (such as lipase and protease) production, which prevented the breakdown of proteins and fats and the release of alkaline compounds and pH increase finally. The results of Fadıloğlu and Emir Çoban (2018) showed that the chitosan‐sumac edible film prevented pH increase in the fish fillets compared to the chitosan film. According to their results, the pH of the samples decreased on the third day of storage due to the dissolution of carbon dioxide in the fish fillet, and then it increased due to the volatile compounds produced, such as ammonia and trimethylamine, by the intracellular or microbial enzymes. Haghighi and Yazdanpanah (2020) (chitosan film containing cinnamon and tea extract), Tavakkoli, Bazargani‐Gilani and Pajohi‐Alamoti (2020) (Arabic gum coating containing tomato pomace extract), Bazargani‐Gilani and Pajohi‐Alamoti (2020) (sodium alginate coating containing resveratrol), Utami et al. (2014) (cassava starch‐based edible coating enriched with *Kaempferia rotunda* and *Curcuma xanthorrhiza* essential oil) and Utami, Nursiwi, and Wohon (2018) (tapioca‐based edible coating enriched with nisin) observed the positive effects of the used edible films and coating in the lowering the bacterial populations and followed the pH decrease in the cold‐stored fish fillets, which was in accordance with our findings.

#### 3.7.2 Thiobarbituric Acid Reactive Substances (TBARS)

Fish flesh is rich in polyunsaturated fatty acids (PUFA), which makes it very sensitive to lipid oxidation. Lipid oxidation leads to the off‐odor and taste in fish flesh (Fadıloğlu and Emir Çoban 2018). Figure 9b illustrates the TBARS values of the cold‐stored trout fillets. During 12 days of the storage of trout fillets at 4°C, lipid oxidation occurred in the samples, which caused TBARS increase in them. The increase intensity was significantly (*p* ≤ 0.05) different among the studied treatments so that the control group was quickly oxidized on the third day and spoiled with a value of 1.077 mg MDA/kg of the fish in the sixth day of the storage time. Considering the TBARS level of 0.5–1 mg MDA/kg fish indicates the spoilage initiation, the control samples showed the amount of 0.67 mg MDA/kg of the fish on the third day of the storage period. While the TBARS values of the other groups were less than 0.5 mg MDA/kg of the fish at this time. According to the obtained results, the groups containing the BRPE significantly (*p* ≤ 0.05) prevented the lipid oxidation and TBARS increase that can be related to their antioxidant compounds, such as betalanin. The TBARS values of the CP‐BRPE‐nAg and CP‐BRPE treatments were 0.705 and 0.907 mg MDA/kg of the fish at the end of the storage period, which is significantly (*p* ≤ 0.05) lower than the CP‐nAg (1.544 mg MDA/kg of fish) and CP (1.972 mg MDA/kg of fish) groups, whose TBARS values exceeded 1 mg MDA/kg of the fish (Figure 9b). In agreement with our results, Fadıloğlu and Emir Çoban (2018) showed the antioxidant activity and oxygen barrier property of the chitosan film containing sumac extract, followed by a low TBARS value in the packaged fish fillets during the refrigerated storage period. The results of Yavuzer, Özogul, and Özogul (2020), Tavakkoli, Bazargani‐Gilani, and Pajohi‐Alamoti (2020) and Haghighi and Yazdanpanah (2020) also showed the positive effects of different edible films and coatings containing natural antioxidant compounds in preventing the TBARS value increase in meat products, which were in accordance with our results.

#### 3.7.3 Total Volatile Basic Nitrogen (TVBN)

Due to the microbial growth and proteolytic enzymes in the fish flesh, proteins are broken and volatile nitrogenous compounds are released. Volatile nitrogenous compounds are tasteless products that result from the microbial and enzymatic degradation of protein‐rich foods and include ammonia, dimethylamine, trimethylamine, etc., which may also change the pH of the product (Wu et al. 2019). In the freshwater fish, the amount of TVBN value around 21–22 mg/100 g of the fish muscle is the acceptable limit, and more than 25 mg/100 g is unconsumable. Figure 9c represents the TVBN values of the studied treatments during the storage period. The initial TVBN value of all groups was around 11.06–11.22 mg/100 g, and then an increasing trend in their TVBN values was observed during the storage time. So, the TVBN value of the control sample quickly increased, and the CP and CP‐BRPE groups were in the next ranks, respectively. All samples showed TVBN values less than 21 mg/100 g until Day 3. On Day 6, the amount of TVBN in the control sample (28.7 mg/100 g) exceeded the allowable limit, which indicates that the fish is inedible. On the ninth day, CP and CP‐BRE groups had TVBN values of 25.9 and 23.9 mg/100 g, respectively. The lowest TVBN values belonged to the CP‐BRPE‐nAg (22.1 mg/100 g of fish muscle) and CP‐nAg (25.7 mg/100 g of fish muscle) groups, respectively at the end of the storage period. The presence of silver nanoparticles and BRPE prevented the microbial growth, followed by the low‐volatile compound production. Tavakkoli, Bazargani‐Gilani, and Pajohi‐Alamoti (2020) reported the positive effects of Arabic gum coating containing tomato residuum extract and dill essential oil on the trout fillet in the decrease of TVBN value, which indicated the efficiency of the used coating in inhibiting the fish spoilage. The results of Haghighi and Yazdanpanah (2020) (chiton‐based edible film containing cinnamon and tea extract), Wu et al. (2019) (chiton‐based edible film containing black rice extract), and Fadıloğlu and Emir Çoban (2018) (chiton‐based edible film containing sumac extract) in lowering the TVBN values of the packaged fish fillets were in accordance with our findings.

### 3.8 Colorimetric Analysis of the Films

**FIGURE 10:**
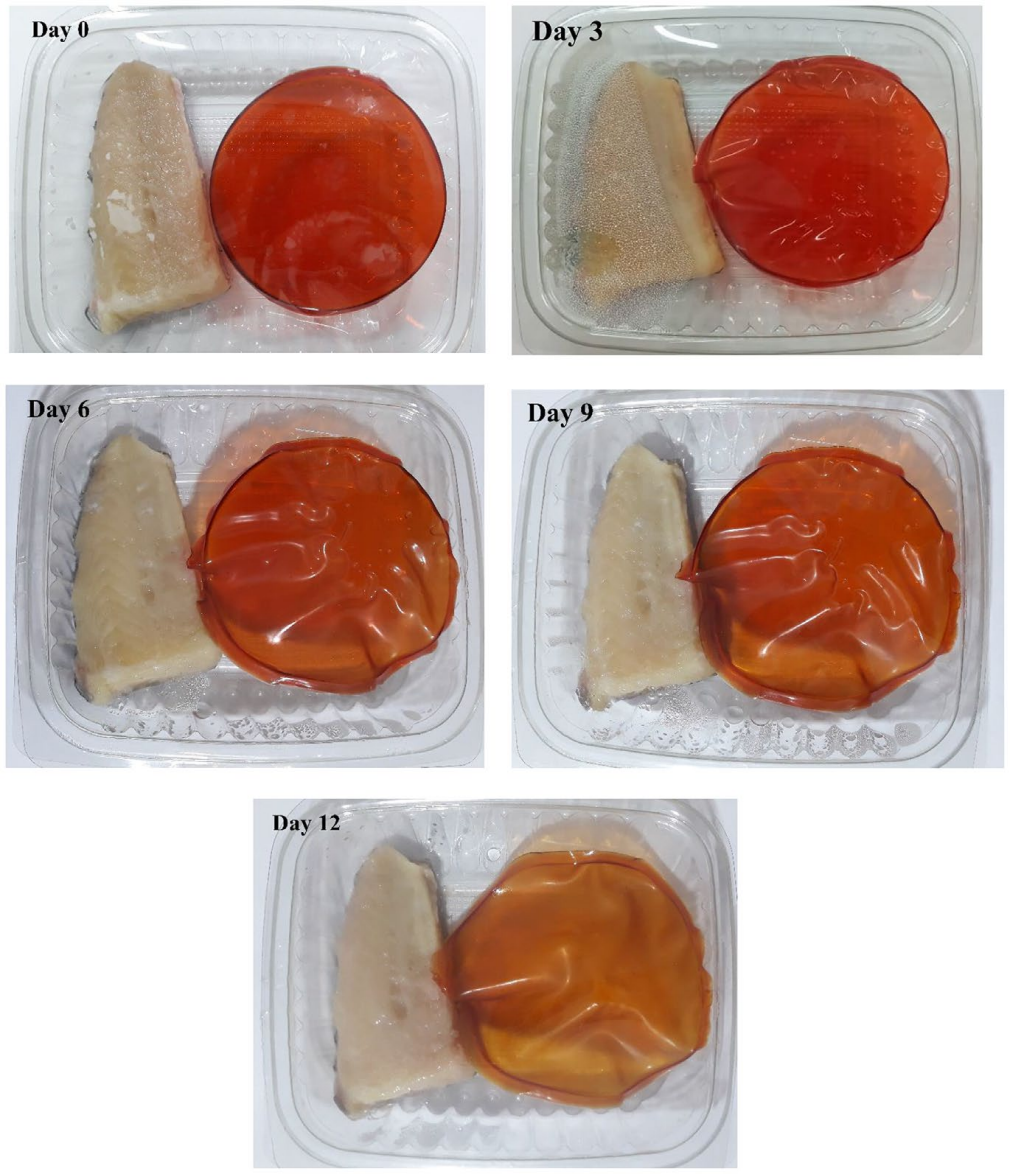
Color response of the CP‐BRPE film during cold storage of the packaged rainbow trout fillets.

The CP‐BRPE treatment was considered for the colorimetric analysis in 0, 3, 6, 9, and 12 days. Three color indexes of L*, a*, and b* of the CP‐BRPE film were analyzed, and the obtained findings are depicted in Table 3. Also, the discolored film images are illustrated in Figure 10. After 3 storage days of the fish fillets, the flat uniform film was wrinkled and deformed, which can be related to the absorption of the produced gases and water vapor of the fillets. This can be related to the gas‐sensitivity of the designed film. By increasing the storage period and appearance of the spoiling symptoms in the trout fillets, the deformed film discolored to yellow so that the film color changed to yellow at the end of the storage period, as proved by the redness index (a*) decrease and the yellowness index (b*) increase. Therefore, the film discoloring was coordinated to the microbial, chemical, and sensory analyses of the cold‐stored trout fillets. Smart packaging undergoes the changes in color, morphology, and etc. following the food spoilage and can provide information about the food condition for the consumers. The release of the nitrogenous compounds as a result of the food spoilage causes pH changes in food that lead to the color change of their smart packaging (Chen et al. 2021). Wu et al. (2019) concluded that the color of chitosan film containing black rice extract changed from purple to gray due to the packaged shrimp spoilage. Chen et al. (2021) reported the color change of chitosan film containing red cabbage extract from purple to yellow by increasing the TVBN value, followed by the pH change of the packaged long tail fish and shrimp. Guo et al. (2021) observed a red to brown color change in pectin film containing red beetroot extract in cold‐stored red meat. Yao et al. (2020) also found the color change of chitosan film containing cactus betalains extract from purple to yellow by increasing the TVBN value in the stored shrimp.

**TABLE 3:** Colorimetric features of the CP‐BRPE film during refrigerated storage of trout fillets.

Day	L*	a*	b*
0	48.43 ± 0.16^d^	57.64 ± 0.31^a^	15.39 ± 0.07^e^
3	49.94 ± 0.32^c^	40.54 ± 0.52^b^	22.76 ± 0.07^d^
6	51.43 ± 0.39^b^	28.81 ± 0.59^c^	29.54 ± 0.40^c^
9	52.80 ± 0.36^a^	24.73 ± 0.93^d^	35.35 ± 0.46^b^
12	53.45 ± 0.36^a^	16.48 ± 0.44^e^	39.17 ± 0.74^a^

*Note:* Different letters indicate a statistically significant difference (*p* ≤ 0.05) for the same column.

### 3.9 Sensory Analysis

**FIGURE 11:**
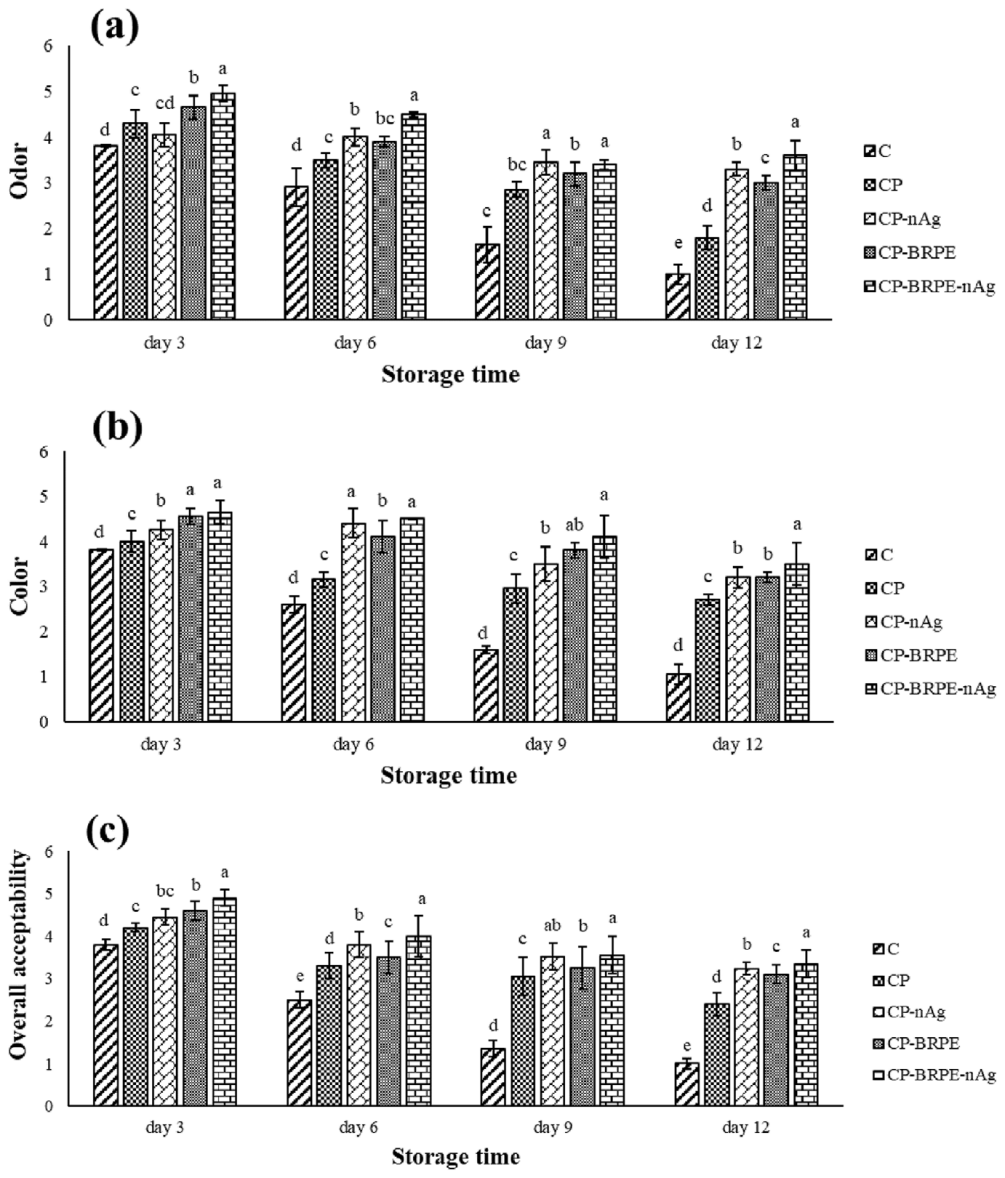
(a–c) The changes in the sensory attributes (a: odor, b: color, and c: overall acceptability) of the studied treatments during storage period. Treatments: C (control, fillets without packaging), CP (fillets packaged with chitosan‐polyvinyl alcohol), CP‐nAg (fillets packaged with chitosan‐polyvinyl alcohol‐nano silver particles), CP‐BRPE (fillets packaged with chitosan‐polyvinyl alcohol‐beetroot peel extract), and CP‐BRPE‐nAg (fillets packaged with chitosan‐polyvinyl alcohol‐beet root peel extract‐nano silver particles). Different letters indicate a statistically significant difference (*p* ≤ 0.05) for the same day.

Figure 11a–c illustrates the sensory features (odor, color, and overall acceptability) of the packaged trout fillets during the storage period. The control sample received the lowest scores in all studied features during the storage time and achieved the unacceptable score (< 3) in three sensory features from the 6th day of storage. The highest scores in odor, color, and overall acceptability belonged to the CP‐BRPE‐nAg treatment, and CP‐nAg, CP‐BRPE, and the CP groups were in the next ranks, respectively. It seems that nAg and BRPE could preserve the sensory features of the trout fillets by decreasing the microbial and chemical changes of them during the refrigerated storage period. The results of Tavakkoli, Bazargani‐Gilani, and Pajohi‐Alamoti (2020) (Arabic gum coating containing tomato residuum extract), Bazargani‐Gilani and Pajohi‐Alamoti (2020) (Sodium alginate coating containing resveratrol), and Fadıloğlu and Emir Çoban (2018) (chitoan‐based edible coating containing sumac extract) showed that the enriched edible coatings by herbal extracts prevented the unpleasant sensory attributes, such as odor, color, and overall acceptability, by lowering the microbial growth and chemical reactions in the cold‐stored fish fillets, which is in agreement with our results. Ahmed et al. (2022) showed that corn starch‐gelatin‐silver nanoparticles film preserved the sensory characteristics of the packaged chicken breast meat compared to the unpackaged samples. They concluded that the unpackaged chicken fillet spoiled on the 4th day of refrigerated storage, while the packaged sample by corn starch‐gelatin‐silver nanoparticles film had a higher score on the same day.

## 4 Conclusion

It is concluded that as a food waste, BRPE showed an appropriate color response against a pH range of 3–12 and depicted the trout fillet spoilage in CP film. Therefore, BRPE is a pH‐sensitive compound and can be considered as a natural, cost‐effective pH indicator in intelligent packaging. Also, silver nanoparticles were biosynthesized using chitosan and were proved by UV–Visible spectroscopy, XRD, optical microscope, and SEM images. Furthermore, CP film containing nAg and BRPE improved the shelf life of the refrigerated rainbow trout fillets during 12 days of the storage period. The antioxidant properties of BRPE, along with the antimicrobial features of nAg, significantly (*p* ≤ 0.05) strengthened the preservative effects of the CP films in the cold‐stored trout fillets. So, the CP‐BRPE‐nAg film was the strongest group in shelf life improvement of the trout fillets among the other films under the refrigerated conditions. The usage of the BRPE as a pH indicator in the other types of the edible films and coatings in various foods is proposed for the future studies.

## Author Contributions

**Fatemeh Ghanbar Soleiman Abadi:** data curation (equal), formal analysis (equal), investigation (equal), methodology (equal), software (equal), writing – original draft (equal). **Behnaz Bazargani‐Gilani:** conceptualization, supervision, methodology, formal analysis, writing – original draft, investigation, funding acquisition. **Aryou Emamifar:** conceptualization (equal), funding acquisition (equal), methodology (equal), software (equal), validation (equal), writing – review and editing (equal). **Alireza Nourian:** funding acquisition (equal), investigation (equal), methodology (equal), software (equal), writing – review and editing (equal).

## Ethics Statement

This study does not involve any human or animal testing.

## Conflicts of Interest

The authors declare no conflicts of interest.

## Data Availability

The data that support the findings of this study are available on request from the corresponding author.

## References

[fsn34605-bib-0001] Ahmed, O. , F. Khalafalla , F. Ali , and A. Hassan . 2022. “Biodegradable Antimicrobial Films Incorporated With Silver Nanoparticles Inhibit the Growth of Multiple Drug‐Resistant *Staphylococcus aureus* Experimentally Inoculated in Chicken Fillets.” Journal of Veterinary Medical Research 29, no. 2: 66–71.

[fsn34605-bib-0002] Ali, A. , and S. Ahmed . 2021. “Eco‐Friendly Natural Extract Loaded Antioxidative Chitosan/Polyvinyl Alcohol Based Active Films for Food Packaging.” Heliyon 7, no. 3: e06550. 33851050 10.1016/j.heliyon.2021.e06550 PMC8024601

[fsn34605-bib-0003] ASTM . 2012. Standard Test Method for Tensile Properties of Thin Plastic Sheeting D882‐12. United states: ASTM, Annual Book of American Standard Testing Methods.

[fsn34605-bib-0004] Bazargani‐Gilani, B. , and M. Pajohi‐Alamoti . 2020. “The Effects of Incorporated Resveratrol in Edible Coating Based on Sodium Alginate on the Refrigerated Trout ( *Oncorhynchus mykiss* ) Fillets' Sensorial and Physicochemical Features.” Food Science and Biotechnology 29, no. 2: 207–216. 32064129 10.1007/s10068-019-00661-1 PMC6992829

[fsn34605-bib-0005] Bazargani‐Gilani, B. , M. Pajohi‐Alamoti , P. Hassanzadeh , and M. Raeisi . 2021. “Impacts of Carboxymethyl Cellulose Containing Propolis Extract on the Shelf Life of Trout Fillets.” Archives of Hygiene Sciences 10, no. 2: 117–132.

[fsn34605-bib-0006] Blois, M. S. 1958. “Antioxidant Determinations by the Use of a Stable Free Radical.” Nature 181, no. 4617: 1199–1200.

[fsn34605-bib-0007] Brannan, R. G. 2008. “Effect of Grape Seed Extract on Physicochemical Properties of Ground, Salted, Chicken Thigh Meat During Refrigerated Storage at Different Relative Humidity Levels.” Journal of Food Science 73, no. 1: C36–C40. 18211347 10.1111/j.1750-3841.2007.00588.x

[fsn34605-bib-0008] Chen, M. , T. Yan , J. Huang , Y. Zhou , and Y. Hu . 2021. “Fabrication of Halochromic Smart Films by Immobilizing Red Cabbage Anthocyanins Into Chitosan/Oxidized‐Chitin Nanocrystals Composites for Real‐Time Hairtail and Shrimp Freshness Monitoring.” International Journal of Biological Macromolecules 179: 90–100. 33636274 10.1016/j.ijbiomac.2021.02.170

[fsn34605-bib-0009] Chhikara, N. , K. Kushwaha , P. Sharma , Y. Gat , and A. Panghal . 2019. “Bioactive Compounds of Beetroot and Utilization in Food Processing Industry: A Critical Review.” Food Chemistry 272: 192–200. 30309532 10.1016/j.foodchem.2018.08.022

[fsn34605-bib-0010] Fadıloğlu, E. E. , and Ö. Emir Çoban . 2018. “Effects of Chitosan Edible Coatings Enriched With Sumac on the Quality and the Shelf Life of Rainbow Trout ( *Oncorhynchus mykiss* , Walbaum, 1792) Fillets.” Journal of Food Safety 38, no. 6: e12545.

[fsn34605-bib-0011] Fernández, K. , E. Aspe , and M. Roeckel . 2009. “Shelf‐Life Extension on Fillets of Atlantic Salmon ( *Salmo salar* ) Using Natural Additives, Superchilling and Modified Atmosphere Packaging.” Food Control 20, no. 11: 1036–1042.

[fsn34605-bib-0012] Fu, Y. , J. Shi , S.‐Y. Xie , T.‐Y. Zhang , O. P. Soladoye , and R. E. Aluko . 2020. “Red Beetroot Betalains: Perspectives on Extraction, Processing, and Potential Health Benefits.” Journal of Agricultural and Food Chemistry 68, no. 42: 11595–11611. 33040529 10.1021/acs.jafc.0c04241

[fsn34605-bib-0013] Gasti, T. , S. Dixit , O. J. D'Souza , et al. 2021. “Smart Biodegradable Films Based on Chitosan/Methylcellulose Containing *Phyllanthus reticulatus* Anthocyanin for Monitoring the Freshness of Fish Fillet.” International Journal of Biological Macromolecules 187: 451–461. 34324903 10.1016/j.ijbiomac.2021.07.128

[fsn34605-bib-0014] Ghomi, M. R. , M. Haghi , M. Mohseni , and M. Ghane . 2021. “Expression and Comparison of Growth Gene (IGF‐1) in Native and Non‐Native Rainbow Trout ( *Oncorhynchus mykiss* ) in Three Different Sizes.” Journal of Animal Physiology and Development 14, no. 4: 101–113.

[fsn34605-bib-0015] Guo, Z. , X. Ge , W. Li , L. Yang , L. Han , and Q.‐L. Yu . 2021. “Active‐Intelligent Film Based on Pectin From Watermelon Peel Containing Beetroot Extract to Monitor the Freshness of Packaged Chilled Beef.” Food Hydrocolloids 119: 106751.

[fsn34605-bib-0016] Haghighi, M. , and S. Yazdanpanah . 2020. “Chitosan‐Based Coatings Incorporated With Cinnamon and Tea Extracts to Extend the Fish Fillets Shelf Life: Validation by FTIR Spectroscopy Technique.” Journal of Food Quality 2020: 8865234.

[fsn34605-bib-0017] Hassanisaadi, M. , A. H. S. Bonjar , A. Rahdar , R. S. Varma , N. Ajalli , and S. Pandey . 2022. “Eco‐Friendly Biosynthesis of Silver Nanoparticles Using *Aloysia citrodora* Leaf Extract and Evaluations of Their Bioactivities.” Materials Today Communications 33: 104183.

[fsn34605-bib-0018] ICMSF . 1986. Sampling Plans for Microorganisms in Foods. International Commission on Microbiological Specifications for Foods. London: Blackwell Scientific Publications.

[fsn34605-bib-0019] Kailasa, S. K. , T.‐J. Park , J. V. Rohit , and J. R. Koduru . 2019. “Antimicrobial Activity of Silver Nanoparticles.” In Nanoparticles in Pharmacotherapy, 461–484. United states: Elsevier.

[fsn34605-bib-0020] Koosha, M. , and S. Hamedi . 2019. “Intelligent Chitosan/PVA Nanocomposite Films Containing Black Carrot Anthocyanin and Bentonite Nanoclays With Improved Mechanical, Thermal and Antibacterial Properties.” Progress in Organic Coatings 127: 338–347.

[fsn34605-bib-0021] Kumar, B. , K. Smita , E. Sánchez , A. Debut , and L. Cumbal . 2021. “ *Plukenetia volubilis* L. Seed Flour Mediated Biofabrication and Characterization of Silver Nanoparticles.” Chemical Physics Letters 781: 138993.

[fsn34605-bib-0022] Madian, N. G. , and N. Mohamed . 2020. “Enhancement of the Dynamic Mechanical Properties of Chitosan Thin Films by Crosslinking With Greenly Synthesized Silver Nanoparticles.” Journal of Materials Research and Technology 9, no. 6: 12970–12975.

[fsn34605-bib-0023] Maqbool, H. , M. P. Safeena , Z. Abubacker , M. Azhar , and S. Kumar . 2021. “Effect of Beetroot Peel Dip Treatment on the Quality Preservation of Deccan Mahseer ( *Tor khudree* ) Steaks During Frozen Storage (−18°C).” LWT 151: 112222.

[fsn34605-bib-0024] Medeiros Silva, V. D. , M. C. Coutinho Macedo , C. G. Rodrigues , A. Neris dos Santos , A. C. Loyola , and C. A. Fante . 2020. “Biodegradable Edible Films of Ripe Banana Peel and Starch Enriched With Extract of *Eriobotrya japonica* Leaves.” Food Bioscience 38: 100750.

[fsn34605-bib-0025] Mohamed, N. , and N. G. Madian . 2020. “Evaluation of the Mechanical, Physical and Antimicrobial Properties of Chitosan Thin Films Doped With Greenly Synthesized Silver Nanoparticles.” Materials Today Communications 25: 101372.

[fsn34605-bib-0026] Nwankwo, C. C. 2021. “Antimicrobial and Antihelminthic Activities of Beetroot Plant.” GSC Biological and Pharmaceutical Sciences 15, no. 3: 93–101.

[fsn34605-bib-0027] Oyaizu, M. 1986. “Studies on Products of Browning Reaction Antioxidative Activities of Products of Browning Reaction Prepared From Glucosamine.” Japanese Journal of Nutrition and Dietetics 44, no. 6: 307–315.

[fsn34605-bib-0028] Pandey, V. K. , S. N. Upadhyay , K. Niranjan , and P. K. Mishra . 2020. “Antimicrobial Biodegradable Chitosan‐Based Composite Nano‐Layers for Food Packaging.” International Journal of Biological Macromolecules 157: 212–219. 32339572 10.1016/j.ijbiomac.2020.04.149

[fsn34605-bib-0029] Pikul, J. , D. E. Leszczynski , and F. A. Kummerow . 1989. “Evaluation of Three Modified TBA Methods for Measuring Lipid Oxidation in Chicken Meat.” Journal of Agricultural and Food Chemistry 37, no. 5: 1309–1313.

[fsn34605-bib-0030] Roy, S. , and J. W. Rhim . 2021. “Anthocyanin Food Colorant and Its Application in pH‐Responsive Color Change Indicator Films.” Critical Reviews in Food Science and Nutrition 61, no. 14: 2297–2325. 32543217 10.1080/10408398.2020.1776211

[fsn34605-bib-0031] Salamatullah, A. M. , K. Hayat , M. S. Alkaltham , et al. 2021. “Bioactive and Antimicrobial Properties of Oven‐Dried Beetroot (Pulp and Peel) Using Different Solvents.” PRO 9, no. 4: 588.

[fsn34605-bib-0032] Selvaraj, S. , R. Thangam , and N. N. Fathima . 2018. “Electrospinning of Casein Nanofibers With Silver Nanoparticles for Potential Biomedical Applications.” International Journal of Biological Macromolecules 120: 1674–1681. 30268753 10.1016/j.ijbiomac.2018.09.177

[fsn34605-bib-0033] Šeremet, D. , K. Durgo , S. Jokić , et al. 2020. “Valorization of Banana and Red Beetroot Peels: Determination of Basic Macrocomponent Composition, Application of Novel Extraction Methodology and Assessment of Biological Activity In Vitro.” Sustainability 12, no. 11: 4539.

[fsn34605-bib-0034] Shah, A. , I. Hussain , and G. Murtaza . 2018. “Chemical Synthesis and Characterization of Chitosan/Silver Nanocomposites Films and Their Potential Antibacterial Activity.” International Journal of Biological Macromolecules 116: 520–529. 29758310 10.1016/j.ijbiomac.2018.05.057

[fsn34605-bib-0035] Shankar, S. , D. Khodaei , and M. Lacroix . 2021. “Effect of Chitosan/Essential Oils/Silver Nanoparticles Composite Films Packaging and Gamma Irradiation on Shelf Life of Strawberries.” Food Hydrocolloids 117: 106750.

[fsn34605-bib-0036] Singleton, V. L. , R. Orthofer , and R. M. Lamuela‐Raventós . 1999. “Analysis of Total Phenols and Other Oxidation Substrates and Antioxidants by Means of Folin‐Ciocalteu Reagent.” In Methods in Enzymology, vol. 299, 152–178. United states: Elsevier.

[fsn34605-bib-0037] Tavakkoli, E. , B. Bazargani‐Gilani , and M. Pajohi‐Alamoti . 2020. “The Impacts of Tomato Residuum Extract With Arabic Gum and Dill Essential Oil on the Shelf Life Improvement of Trout Fillets Stored at Chilly Condition.” Journal of Food Safety 40, no. 4: e12812.

[fsn34605-bib-0038] Utami, R. , E. Kawiji Nurhartadi , A. Yusuf Trisna Putra , and A. Setiawan . 2014. “The Effect of Cassava Starch‐Based Edible Coating Enriched With *Kaempferia rotunda* and *Curcuma xanthorrhiza* Essential Oil on Refrigerated Patin Filets Quality.” International Food Research Journal 21, no. 1: 413–419.

[fsn34605-bib-0039] Utami, R. , A. Nursiwi , and N. Wohon . 2018. “The Effect of Tapioca‐Based Edible Coating Enriched With Nisin on Quality of Patin ( *Pangasius hypophthalmus* ) Fillet During Cold Storage.” Jurnal Teknologi 80, no. 4: 111–116.

[fsn34605-bib-0040] Wu, C. , J. Sun , P. Zheng , et al. 2019. “Preparation of an Intelligent Film Based on Chitosan/Oxidized Chitin Nanocrystals Incorporating Black Rice Bran Anthocyanins for Seafood Spoilage Monitoring.” Carbohydrate Polymers 222: 115006. 31320067 10.1016/j.carbpol.2019.115006

[fsn34605-bib-0041] Yan, J. , R. Cui , Y. Qin , L. Li , and M. Yuan . 2021. “A pH Indicator Film Based on Chitosan and Butterfly Pudding Extract for Monitoring Fish Freshness.” International Journal of Biological Macromolecules 177: 328–336. 33621573 10.1016/j.ijbiomac.2021.02.137

[fsn34605-bib-0042] Yao, X. , H. Hu , Y. Qin , and J. Liu . 2020. “Development of Antioxidant, Antimicrobial and ammonia‐Sensitive Films Based on Quaternary Ammonium Chitosan, Polyvinyl Alcohol and Betalains‐Rich Cactus Pears ( *Opuntia ficus‐indica* ) Extract.” Food Hydrocolloids 106: 105896.

[fsn34605-bib-0043] Yavuzer, E. , F. Özogul , and Y. Özogul . 2020. “Impact of Icing With Potato, Sweet Potato, Sugar Beet, and Red Beet Peel Extract on the Sensory, Chemical, and Microbiological Changes of Rainbow Trout ( *Oncorhynchus mykiss* ) Fillets Stored at (3°C ±1°C).” Aquaculture International 28, no. 1: 187–197.

[fsn34605-bib-0044] Yong, H. , J. Liu , Y. Qin , R. Bai , X. Zhang , and J. Liu . 2019. “Antioxidant and pH‐Sensitive Films Developed by Incorporating Purple and Black Rice Extracts Into Chitosan Matrix.” International Journal of Biological Macromolecules 137: 307–316. 31276717 10.1016/j.ijbiomac.2019.07.009

[fsn34605-bib-0045] Yong, H. , X. Wang , R. Bai , Z. Miao , X. Zhang , and J. Liu . 2019. “Development of Antioxidant and Intelligent pH‐Sensing Packaging Films by Incorporating Purple‐Fleshed Sweet Potato Extract Into Chitosan Matrix.” Food Hydrocolloids 90: 216–224.

[fsn34605-bib-0046] Yousef, A. E. , and C. Carlstrom . 2003. Food Microbiology: A Laboratory Manual, 115–117. United states: John Wiley & Sons.

